# Circulating Lactonase Activity but Not Protein Level of PON-1 Predicts Adverse Outcomes in Subjects with Chronic Kidney Disease

**DOI:** 10.3390/jcm8071034

**Published:** 2019-07-15

**Authors:** Chrysan J. Mohammed, Yanmei Xie, Pamela S. Brewster, Subhanwita Ghosh, Prabhatchandra Dube, Tiana Sarsour, Andrew L. Kleinhenz, Erin L. Crawford, Deepak Malhotra, Richard W. James, Philip A. Kalra, Steven T. Haller, David J. Kennedy

**Affiliations:** 1 Department of Medicine, University of Toledo College of Medicine and Life Sciences, Toledo, OH 43614, USA; 2 Department of Internal Medicine, Geneva University Hospital, 1205 Geneva, Switzerland; 3 Department of Renal Medicine, Salford Royal Hospital, Stott Lane, Salford, Greater Manchester M6 8HD, UK

**Keywords:** paraoxonase, lactonase activity, chronic kidney disease, clinical outcomes

## Abstract

The burden of cardiovascular disease and death in chronic kidney disease (CKD) outpaces that of the other diseases and is not adequately described by traditional risk factors alone. Diminished activity of paraoxonase (PON)-1 is associated with increased oxidant stress, a common feature underlying the pathogenesis of CKD. We aimed to assess the prognostic value of circulating PON-1 protein and PON lactonase activity on adverse clinical outcomes across various stages and etiologies of CKD. Circulating PON-1 protein levels and PON lactonase activity were measured simultaneously in patients with CKD as well as a cohort of apparently healthy non-CKD subjects. Both circulating PON-1 protein levels and PON lactonase activity were significantly lower in CKD patients compared to the non-CKD subjects. Similarly, across all stages of CKD, circulating PON-1 protein and PON lactonase activity were significantly lower in patients with CKD compared to the non-CKD controls. Circulating PON lactonase activity, but not protein levels, predicted future adverse clinical outcomes, even after adjustment for traditional risk factors. The combination of lower circulating protein levels and higher activity within the CKD subjects were associated with the best survival outcomes. These findings demonstrate that diminished circulating PON lactonase activity, but not protein levels, predicts higher risk of future adverse clinical outcomes in patients with CKD.

## 1. Introduction

Chronic kidney disease (CKD) affects 30 million people in the United States [1], and it is the twelfth most common cause of death worldwide, accounting for over one million deaths [2]. The burden of cardiovascular morbidity and mortality, accompanying CKD, significantly outpaces that of other diseases [3,4,5]. However, this significant morbidity and mortality is not explained by traditional risk factors alone. While the molecular pathways that drive CKD progression are multifactorial, there are several lines of evidence supporting the central role of dyslipidemias and dysfunctional high-density lipoprotein (HDL) cholesterol in promoting the inflammation and oxidative stress underlying the pathogenesis and the clinical sequela of this disease [6,7,8,9,10,11]. Notably, the influence of dyslipidemia on outcomes in CKD is even more pronounced in African American vs. American Caucasians [12].

Paraoxonases (PON) are a family of hydrolytic enzymes that include three distinct isoforms: PON-1, PON-2, and PON-3. These enzymes are highly conserved, sharing 60–70% nucleic acid homology. While they possess both unique and overlapping functions [13], the antioxidant and anti-inflammatory functions of PONs are well established [14,15,16]. In particular, PON-1 is a calcium dependent enzyme that is primarily produced in the liver, associates with HDL, and is secreted into circulation [17,18]. In fact, PON-1’s association with HDL protects HDL from oxidative modifications and is responsible for much of HDL’s antioxidant, anti-inflammatory, and anti-atherogenic properties, such as protecting low-density lipoprotein (LDL) from oxidation, macrophage cholesterol efflux, and reverse cholesterol transport [19]. These anti-atherogenic mechanisms aid in preventing macrophage cholesterol accumulation and have been the focus of significant research.

Historically, PON derived its name from its ability to hydrolyze paraoxon, an organophosphate contained in some pesticides [20]. Research over the past several decades has revealed numerous other substrates for this enzyme including its ability to hydrolyze arylesterases such as, phenyl acetates [13,18]. More recently the ability of PONs to hydrolyze lactones such as homocysteine thiolactone has been established as the native physiologic activity [21,22]. As such, the lactonase activity has been a focus of PON’s anti-atherosclerotic functions [23,24].

While previous studies investigating the role of PONs in clinical outcomes have mainly focused on non-physiologic measures of PON activity, such as arylesterase and paraoxonase activity, the native physiologic activity of PON’s is that as a lactonase [22]. Importantly, little is known about the relationship of PON lactonase activity to clinical outcomes, especially in the CKD setting. Further, whether diminished circulating PON activity is related to diminished circulating concentrations of PON-1 protein in these settings is unknown. While several studies have reported diminished PON enzymatic activity in CKD patients [9,25], these studies did not simultaneously measure the circulating PON-1 protein levels and the relationship between circulating PON-1 protein and adverse events is unclear. Therefore, we performed the current study to examine the ability of physiologically relevant PON lactonase activity to predict adverse clinical outcomes in the CKD setting and to assess the relationship between the circulating PON-1 protein, PON lactonase activity, and the adverse clinical outcomes in this setting.

## 2. Methods

### 2.1. Study Population

Circulating PON-1 protein level and PON lactonase activity were measured in baseline plasma samples collected from a cohort of 248 patients with CKD, enrolled in the Chronic Renal Insufficiency Standards and Implementation Study (CRISIS), which has subsequently become a part of the larger Salford Kidney Study (SKS). CRISIS was an observational study of outcomes in an all-cause non-dialysis CKD population of 1750 patients recruited in secondary care from Salford, Greater Manchester, United Kingdom [26]. All participants provided written informed consent and were followed for a median of 4.5 years (interquartile range (IQR) 2.9–6.9). Patients without an immediate need for dialysis who were 18 years and older with estimated glomerular filtration rate (eGFR) >10 and <60  mL/min/1.73  m^2^ were eligible to participate. The Modification of Diet in Renal Disease Study and Chronic Kidney Disease Epidemiology Collaboration equations were used to estimate the glomerular filtration rate [27,28,29]. The National Kidney Foundation’s modified Kidney Disease Outcomes Quality Initiative (K/DOQI) classification of CKD [30] was used to classify the CKD stages with stage 2 having eGFR of 60–89 mL/min/1.73 m^2^, stage 3 eGFR of 30–59 mL/min/1.73 m^2^, stage 4 eGFR 15–29 mL/min/1.73 m^2^, and stage 5 eGFR < 15 mL/min/1.73 m^2^. This study was performed in compliance with all regulations and guidelines as approved by the National Health Service Research and Ethics Committee and the University of Toledo Institutional Review Board. Detailed methodology for the CRISIS trial has been previously published [26,28,29] and the baseline characteristics are presented in Table 1. 

In a separate protocol, 33 apparently healthy volunteer participants at the University of Toledo Medical Center (mean age 29.6 ± 9.6 years, mean systolic blood pressure 117.5 ± 10.2, mean diastolic blood pressure 73.7 ± 7.4, 55% female, 81% White, 9% Asian, 3% Black, 7% other) without a history of CKD assessed by the Chronic Kidney Disease Epidemiological creatinine-based estimation of glomerular filtration rate (eGFR) served as non-CKD controls. These participants did not report any active medical conditions at the time of blood draw. The study protocol was approved by the University of Toledo Institutional Review Board and written informed consent was obtained from each of the study participants prior to their participation in the study.

### 2.2. Biochemical Assays

Circulating levels of human total PON-1 were measured in Lithium-heparin plasma by an enzyme-linked immunosorbent assay (ELISA) purchased from R&D Systems (catalog No. DYC5816-5) and performed according to the manufacturer’s recommendations. Samples for ELISA were prepared at 100× dilution in sample diluent purchased from R&D Systems (catalog No. DYC001). The ELISA assay kit contained human total PON-1 capture antibody, detection antibody, PON-1 standard and streptavidin HRP. Additional reagents such as reagent diluent (catalog No. DY995), substrate solution (catalogue No. DY999), and stop solution (catalog No. DY994) were also purchased from R&D systems. The minimum and maximum amount of detectable PON-1 were 0.15 ng/mL and 10 ng/mL, respectively. Western blot was performed independently using a monoclonal antibody to PON-1 [31] to validate the ability of the ELISA to detect the presence of circulating PON-1 protein in plasma (Figure S4). 

Circulating lactonase activity of PON was measured in the patient serum samples with a commercially available fluorometric assay (BioVision Incorporated, catalog # K999-100). Serum PON lactonase activity was calculated as the hydrolytic activity toward a fluorogenic benzopyran-2-one substrate of PON in the presence and absence of a specific PON inhibitor (2-hydroxyquinoline) according to the manufacturer’s protocol.

### 2.3. Statistical Analysis

Continuous data were tested for goodness-of-fit to the normal distribution using the Shapiro–Wilk test. If not normally distributed, the log-transformation of the variable was assessed for normality. Continuous data are presented as mean ± standard deviation (SD) of the untransformed or log-transformed version or, if neither were normally distributed, as median with interquartile range (IQR). Categorical data are presented as frequency and percent. Comparisons between the control group and the combined all-cause CKD group for continuous data were evaluated using two-sample t-tests or Mann–Whitney U rank test. Separate comparisons of circulating PON lactonase activity and PON-1 protein, in addition to a PON lactonase adjusted activity measure derived by dividing the circulating PON-1 protein by the PON lactonase activity, were undertaken between the control and all-cause CKD groups. For categorical variables, the chi-square test or, if the frequency of counts for some factors was low (≤5), Fisher exact test was used to compare the groups. 

Comparisons of continuous data for the CKD cohort stratified by CKD etiology were performed using ANOVA with post-hoc pairwise contrasts using the Tukey–Kramer multiple comparisons test or Kruskal–Wallis test with post hoc Dunn’s multiple comparisons test. Contingency table analysis with chi-square test, or the Fisher exact test for low frequency of counts was used for multi-level categorical variables. The Spearman correlation was performed to determine the relationship between the PON lactonase activity and PON-1 protein levels. Subjects were dichotomized based on the baseline plasma levels of either PON lactonase activity, PON-1 protein, and PON lactonase adjusted activity into high (greater than median) or low (less than or equal to median) groups and were used to predict incident 10-year all-cause mortality risks and non-fatal outcomes based on the cardio-renal events. Subjects were stratified by the combination of both median PON lactonase activity and PON-1 protein in order to evaluate the combined effect of PON lactonase activity and PON-1 protein on CKD progression. Further stratification using quartiles was employed to predict the incident 8-year event risks and to confirm the reliability of median analysis. Eight year event-free survival was used due to few events beyond 8 years. Stepwise logistic regression with PON lactonase activity (high vs. low) as the outcome was used to identify the characteristics significantly related to group membership. Kaplan–Meier estimates with the log-rank statistic were applied to compare the high and low medians for the PON lactonase activity, PON-1 protein, and adjusted PON lactonase activity groups. Cox proportional hazards regression was performed to determine the hazard ratios (HRs) and 95% confidence intervals (CI) for mortality. The analysis was adjusted for traditional risk factors including age, sex, systolic blood pressure, urine protein (log), and myocardial infarction, as well as medication use (beta-blocker and angiotensin-converting enzyme inhibitors, angiotensin II receptor blockers). The proportional hazards assumption tests from the R function cox.zph in the survival package for all the survival models had *p*-values > 0.05 indicating that the null hypothesis of proportional hazards was not rejected. All analyses were performed using R 3.4.2 (www.r-project.org) and statistical significance was defined as a *p*-value < 0.05.

## 3. Results

### 3.1. Subject Characteristics 

Baseline characteristics of the study population are presented in Table 1. The mean age of the CKD cohort was 69 ± 19 years and the mean eGFR was 30.4 ± 25.9 mL/min per 1.73 m^2^. A total of 248 participants had CKD, among whom, 9 (4%) had mild CKD (stage 2), 103 (42%) had moderate CKD (stage 3), 85 (34%) had severe CKD (stage 4), and 51 (20%) had end stage kidney disease (ESKD, stage 5). The etiology of CKD was as follows: 40 (16%) patients had diabetic nephropathy, 16 (7%) patients had adult polycystic kidney disease, 85 (34%) patients had vascular hypertension, 33 (13%) patients had glomerulonephritis/vasculitis, 16 (7%) patients had pyelonephritis, and 58 (23%) patients had other causes of CKD. A total of 216 events were recorded (see Table 1), including both cardiovascular or renal mortality (*n* = 127, 51%), renal replacement therapy (*n* = 66, 27%) or myocardial infarction, congestive heart failure or stroke (*n* = 23, 9%). 

### 3.2. Clinical Characteristics of CKD Subjects

The clinical characteristics of CKD and non-CKD subjects are presented in Tables S1 and S2 respectively. A total of 28 events were recorded for CKD subjects with diabetic nephropathy, 14 events for adult polycystic kidney disease, 59 events for vascular hypertension, 24 events for glomerulonephritis/vasculitis, 5 events for pyelonephritis, and 36 for other causes of CKD. 

### 3.3. Circulating PON-1 Protein and Lactonase Activity Levels across CKD Stages and Etiology

**Figure 1:**
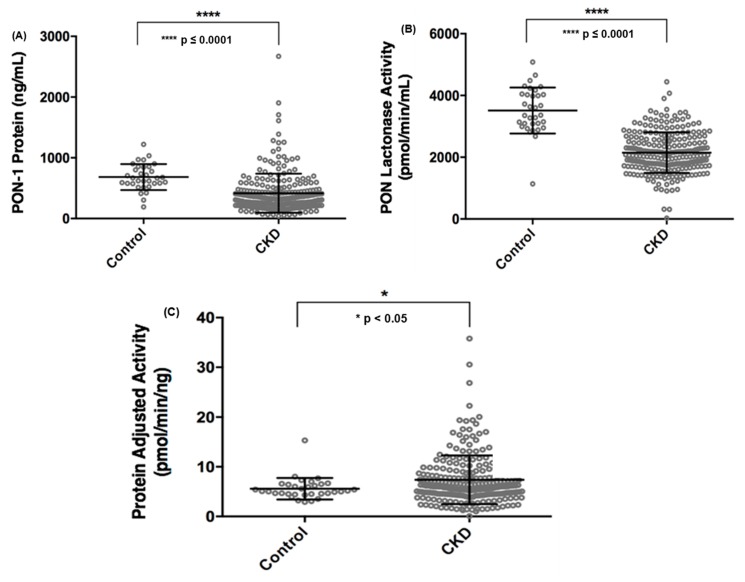
Comparison of circulating paraoxonases-1 (PON-1) protein (**A**), PON lactonase activity (**B**), and PON protein adjusted lactonase activity (**C**) between non-chronic kidney disease (CKD) control subjects and patients with CKD.

We examined the circulating PON-1 protein levels and lactonase activity in the CKD cohort compared to the healthy non-CKD cohort and found that both PON-1 protein level and activity were significantly decreased in CKD subjects compared to the non-CKD study participants (Figure 1A,B). In order to better understand the functional activity of circulating PON-1 protein in the study participants, we adjusted the circulating PON lactonase activity for PON-1 protein level (i.e., circulating PON lactonase activity divided by circulating PON-1 protein level) and noted that PON-1 protein adjusted lactonase activity was increased in the CKD subjects compared to the non-CKD subjects (Figure 1C). We further examined circulating PON-1 protein levels and lactonase activity across stages of CKD. We found that compared to non-CKD subjects both PON-1 protein levels and lactonase activity significantly decreased across CKD stages, except for stage 2 CKD where there was no significant difference in both protein and lactonase activity compared to controls (Figure S1A,B). After adjusting the circulating PON lactonase activity for PON-1 protein level, there was no significant difference across the CKD stages compared to the control subjects (Figure S1C). 

Next, we investigated the circulating PON-1 levels and lactonase activity across various CKD etiologies. Here we found that both circulating protein level and lactonase activity were significantly decreased in all etiologies compared to the non-CKD controls (Figure S2A,B). Interestingly, after adjusting PON lactonase activity for protein level, patients with diabetic nephropathy had significantly higher protein adjusted activity than control, while subjects with other etiologies of CKD had no significant difference in their protein adjusted activity compared to controls (Figure S2C). 

### 3.4. Circulating PON Activity but Not Protein Levels Predicts Adverse Outcomes in CKD

We next examined the baseline PON-1 protein, PON lactonase activity, and PON-1 protein adjusted lactonase activity in order to determine the optimal cut-off levels predictive of survival. In this analysis, individuals with lower circulating PON lactonase activity (≤2073 pmol/min/mL) had worse survival outcomes (hazard ratio 1.66, 95% CI 1.16 to 2.38, *p* < 0.01; Table 2). Lower PON-1 protein adjusted lactonase activity showed similar trends when divided by optimal cut-off (≤6.22 pmol/min/pg; hazard ratio 1.51, 95% CI 1.06 to 2.16, *p* < 0.05; Table 2). Conversely, circulating PON-1 protein levels alone were unable to predict survival in these patients (hazard ratio 0.97, 95% CI 0.68 to 1.37, *p* = NS; Table 2). After adjusting for traditional risk factors such as age, gender, systolic blood pressure, urine protein, prior myocardial infarction, and medication use including β-blocker, angiotensin converting enzyme inhibitors, and angiotensin II receptor blockers, individuals with lower circulating PON lactonase activity and PON adjusted lactonase activity still had worse survival outcomes at 10 years (hazard ratio 1.48, 95% CI 1.02 to 2.14, *p* < 0.05 for PON lactonase activity and hazard ratio 1.55, 95% CI 1.07 to 2.25, *p* < 0.05 for PON-1 protein adjusted lactonase activity; Table 2). 

We further examined this by quartile analysis and found similar trends; the lowest circulating PON lactonase activity quartile (<1732 pmol/min/mL) was predictive of an increased risk of death (hazard ratio 1.76, 95% CI 1.04 to 2.97, *p* = 0.03; Table S3). After adjusting for traditional risk factors and medication use, lower circulating PON lactonase activity still conferred an increased risk of death (hazard ratio 1.92, 95% CI 1.12 to 3.29, *p* = 0.02; Table S3). Subjects in the lowest PON-1 protein adjusted lactonase activity quartile (<4.47 pmol/min/ng) showed similar trends (hazard ratio 1.71, 95% CI 1.03 to 2.85, *p* = 0.04; Table S3); however, after adjusting for traditional risk factors and medication this did not reach statistical significance (hazard ratio 1.51, 95% CI 0.89 to 2.55, *p* = 0.13; Table S3). Again, PON-1 protein levels alone failed to predict survival in this CKD cohort (hazard ratio 0.89, 95% CI 0.54 to 1.46, *p* = 0.64).

### 3.5. Kaplan–Meier Survival Analysis 

**Figure 2:**
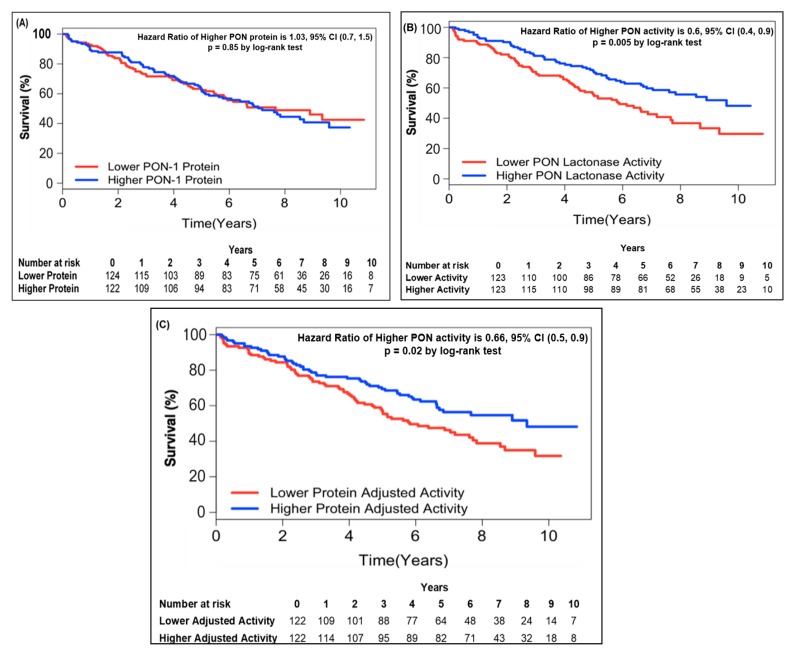
Kaplan–Meier analysis for mortality by PON-1 protein (**A**), PON lactonase activity (**B**), and PON protein adjusted lactonase activity (**C**) in patients with CKD. Patients were stratified according to optimal cut-off as follows: Lower PON-1 protein ≤ 333.4 (ng/mL) and higher PON-1 protein > 333.4 (ng/mL); lower PON lactonase activity ≤ 2073 (pmol/min/mL) and higher PON lactonase activity > 2073 (pmol/min/mL); lower protein adjusted lactonase activity ≤ 6.22 (pmol/min/ng) and higher protein adjusted activity > 6.22 (pmol/min/ng).

**Figure 3:**
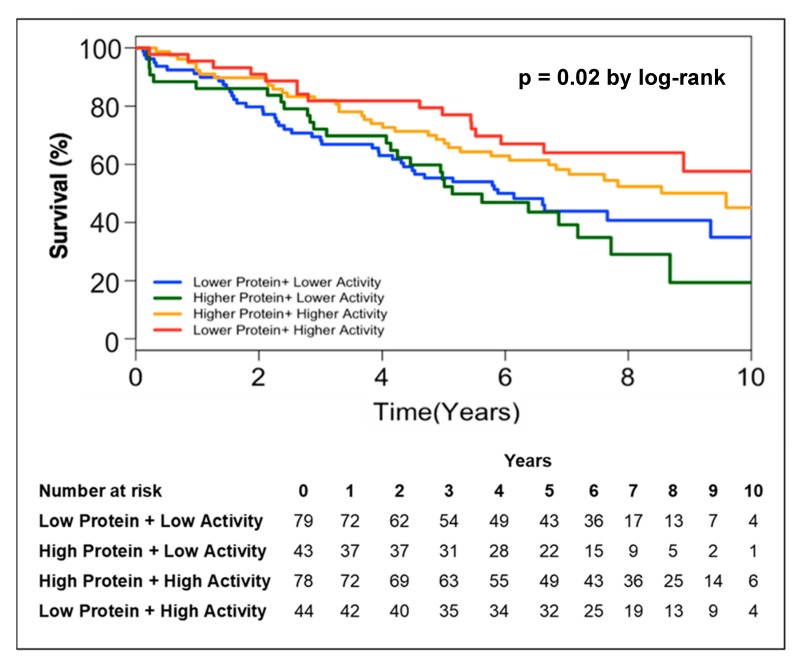
Kaplan–Meier analysis for mortality in patients with CKD stratified by lower protein + higher lactonase activity, higher protein + higher lactonase activity, higher protein + lower lactonase activity, and lower protein + lower lactonase activity.

In order to further address the relationship between circulating PON-1 protein and lactonase activity with mortality, we stratified the subjects into two groups according to high (greater than median) or low (less than or equal to median) circulating PON-1 protein concentration and lactonase activity level. Kaplan–Meier survival analysis demonstrated that lower circulating lactonase activity (≤2073 pmol/min/mL, hazard ratio 0.6, 95% CI 0.4 to 0.9, *p* = 0.005; Figure 2B) but not protein levels (≤333.4 ng/mL, hazard ratio 01.03, 95% CI 0.7 to 1.5, *p* = NS; Figure 2A) was most predictive of future all-cause mortality. After adjusting the circulating PON lactonase activity for protein level, the lower PON-1 protein adjusted lactonase activity still conferred a worse survival outcome (≤6.22 pmol/min/ng, hazard ratio 0.66, 95% CI 0.5 to 0.9, *p* = 0.02; Figure 2C). Kaplan–Meier survival analyses demonstrated that the combination of lower circulating PON-1 protein levels (≤333.4 ng/mL) and higher circulating PON lactonase activity (>2073 pmol/min/mL) within CKD subjects were associated with the highest survival rates compared to those with higher circulating protein levels (>333.4 ng/mL) and lower activity (≤2073 pmol/min/mL, Figure 3).

### 3.6. Factors That Predict PON Lactonase Activity 

We next sought to investigate the clinical characteristics that determined the circulating PON lactonase activity in the CKD setting. First, we examined the relationship between circulating protein and lactonase activity in the non-CKD group and found that circulating PON lactonase activity correlated with the protein levels (*r* = 0.36, *p* = 0.04; Figure S3A). Similarly, in the CKD cohort circulating PON lactonase activity also correlated with PON-1 protein levels, albeit to a more limited extent (*r* = 0.28, *p* ≤ 0.001, Figure S3B). We then performed a multivariate analysis for predictors of PON lactonase activity in CKD based on the clinical characteristics outlined in Table 1 and found that increased age (estimate 0.96, standard error 0.01, *p* = 0.001) and increased body mass index (estimate 0.94, standard error 0.02, *p* = 0.03) were the best predictors of decreased circulating PON lactonase activity in this population (Table 3). 

## 4. Discussion

In the current study, we demonstrated a significant reduction in both circulating PON-1 protein and PON lactonase activity from a large cohort of CKD patients compared to a reference non-CKD population. These reductions were consistent regardless of the CKD etiology. Our findings also indicate that decreased circulating PON lactonase activity was predictive of future all-cause mortality in this CKD cohort. Interestingly, the circulating PON-1 protein levels did not predict all-cause mortality in these patients. To our knowledge, this is the largest study to examine the relationship between circulating PON-1 protein levels and circulating PON lactonase activity across CKD stages and etiologies. Previous studies reporting PON activity in the CKD setting focused on the non-physiological measures of PON enzymatic activity (e.g., arylesterase or paraoxonase activity). However, this is one of the first studies to measure a physiologically relevant enzymatic activity of PON (i.e., lactonase) [22] across all stages of CKD. Additionally, this is also the first study to investigate the relationship between PON-1 protein levels, PON lactonase activity, and adverse clinical outcomes in subjects with CKD. Our finding of a significant decrease in circulating PON-1 concentration and lactonase activity in CKD subjects are in strong agreement with other studies and has recently been extensively reviewed [32,33]. Indeed, PON-1 concentration and activity (as measured by PON arylesterase and paraoxonase activities) are decreased in patients with mild to moderate CKD. In subjects with CKD, diminished PON activity is correlated with increased aortic stiffness and aortic strain, therefore increasing risk of cardiovascular disease [25]. Similarly, in a large (*n* = 630) study of subjects with mild to moderate CKD, diminished PON arylesterase activity predicted an increased risk of developing adverse cardiac events [9]. PON activity is also diminished in patients with ESKD [34,35,36,37,38]. In a small study of hemodialyzed (*n* = 108) and renal transplant patients (*n* = 78), significantly diminished lactonase and paraoxonase activity was observed in both patient groups compared to healthy controls. Additionally, in this same study an increase in PON lactonase and paraoxonase activity was associated with higher HDL levels in renal transplant patients. It is well established that hemodialysis patients have an increased risk of cardiovascular complications, and major contributors include the fact that these patients experience enhanced oxidative stress, dyslipidemia, and endothelial dysfunction resulting in atherosclerotic changes [39,40,41,42]. Sztanek et al. suggested that lactonase activity may be a novel predictor of cardiovascular risk in ESKD [34]. Interestingly, in post hemodialysis treatments and renal transplantation, PON activity is restored in patients with ESKD [34,36,43]. Furthermore, a study by Ikeda et al. showed that ESKD patients on maintenance hemodialysis have decreased PON-1 concentration and that lower PON-1 concentration associates with worse cardiovascular outcomes [44]. However, while several studies have investigated PON activity levels in CKD, there is a paucity of knowledge regarding the relationship between circulating PON-1 concentrations and physiologically relevant PON lactonase activity across the spectrum of CKD. 

In the current study, we found that neither circulating PON-1 protein level nor PON lactonase activity correlated with CKD stage, which suggests that PON is not merely a marker of decreased GFR in this setting. Importantly, as CKD is not a static condition or defined by a single etiology, in the present study we not only stratified circulating PON-1 protein and lactonase activity by CKD stage but also by etiology and found that circulating PON-1 protein concentration and PON lactonase activity decreases regardless of the CKD etiology. These findings are novel, since this is the first study to determine simultaneously the levels of circulating PON-1 protein and lactonase activity across a wide variety of CKD etiologies. 

We also noted that diminished circulating PON lactonase activity, and not circulating PON-1 protein level, predicted the increased risk of adverse clinical outcomes in this CKD cohort. This finding held true even after adjustment for traditional risk factors such as age, gender, systolic blood pressure, proteinuria, prior myocardial infarction, and other cardiovascular risk factors (i.e., prior cerebrovascular accident or peripheral vascular disease) and medication use. Interestingly, when we adjusted PON lactonase activity per unit of circulating PON-1 protein levels, we noted that CKD patients with higher circulating PON lactonase activity per unit of circulating PON-1 protein demonstrated better event-free survival compared to those patients who had lower circulating PON lactonase activity per unit of circulating PON-1 protein. These findings suggest that the functional status of PON, rather than its concentration alone is most important with respect to the outcomes in the CKD setting. The mechanism whereby decreased circulating PON lactonase activity leads to poor all-cause mortality in CKD is not fully understood. Therefore, strategies aimed at understanding mechanistically what drives lactonase activity in these patients will be useful to improve the outcomes in this patient population which experiences significant morbidity and mortality. 

This discordance between the circulating PON lactonase activity and the protein level suggests several possibilities that are not mutually exclusive. It is possible that in the CKD setting there may be factors that are responsible for inhibiting normal PON function such as uremic toxins [9,45], advance glycation end products (AGE) [46], and acrolein [47,48]. PON may also undergo modifications induced by oxidative or nitrative stress, and post translational modifications such as carbamylation, in the CKD setting which render it less biologically active or dysfunctional [49,50]. This possibility is supported by a previous study which demonstrates that carbamylation of PON-1 in uremic patients was higher than in healthy controls and activity of the HDL-associated enzyme was significantly reduced in uremic subjects compared to controls [49]. It is well established that the activity of myeloperoxidase, a source of reactive oxygen species, is elevated in the milieu of CKD consequently rendering HDL dysfunctional [51,52]. One study in particular found that myeloperoxidase can form a ternary complex with PON and HDL, resulting in the oxidative modification and inactivation of PON-1 [53]. Another possibility is that there are genetic factors that predispose individuals to decreased PON activity. A study in CKD patients with cardiomyopathy reported that the severity of left ventricular hypertrophy and left ventricular dysfunction correlates in a dose dependent manner with the R allele in the PON-1 polymorphism Q192R [54]. The Q192R variant is a characteristic marker of oxidant status, where the R allele has been shown to be less protective against low density lipoprotein oxidation than the Q allele [55]. 

Additionally, CKD is associated with lipid abnormalities and patients typically have low HDL and high triglycerides profiles [56]. Dyslipidemia in the CKD setting is associated with a higher risk of disease progression to ESRD and increased mortality [57]. Dyslipidemia can also result in alterations of PON-1 [58]. A study by Miljkovic et al. demonstrated that HDL fractions isolated from patients with renal disease have decreased PON-1 concentration and activity compared to controls [50]. PON activity is also diminished in HDL from patients with heart failure compared to control, while there was also an increase in the levels of oxidized lipids in these patients [59]. A study involving patients with type IIb hypercholesterolaemia found that there was a significant increase in the PON-1 paraoxoanse activity after three months of statin treatments and increased PON activity associated with lower levels of triglycerides [60]. While the current study was not designed to address these possibilities, they indeed warrant further investigation.

In the multivariate analyses, age and BMI are predictors of circulating PON lactonase activity in CKD subjects. This finding is corroborated by previous studies showing that decrease in PON activity is associated with decrease in age [61,62,63,64] and an increase in BMI [65,66,67,68]. Notably, hemodialysis patients show a significant correlation between PON-1 activity and BMI, where obese patients had significantly lower PON-1 paraoxonase activity compared to controls [69]. The decrease of paraoxonase activity in obese patients has also been associated with increases in lipid hydroperoxides of HDL and LDL [70]. Indeed, it is well known that both older and obese individuals are more susceptible to HDL oxidation and in the CKD setting oxidative stress is salient in the pathogenesis of the disease [61,68].

The disproportionate morbidity and mortality experienced by patients with CKD is not explained by traditional risk factors alone. Therefore, identification of new therapeutic targets in this population is essential. The present study identifies PON lactonase activity as an important predictor of adverse clinical outcomes in the CKD setting. Importantly, PON activity may also be a modifiable risk factor as interventions such as pomegranate juice [71,72], flavonoids [73,74], olive oil [75,76], and fibrates [77] have been shown to increase PON activity and may even provide cardiovascular benefits in patients with renal disease [78,79,80]. Whether these interventions may improve outcomes in CKD is a topic of interest. 

Our study provides evidence highlighting the role of diminished circulating PON lactonase activity, but not circulating PON-1 protein, as an important risk factor associated with adverse clinical outcomes in CKD. Furthermore, our study demonstrates that higher PON lactonase activity is associated with increased survival in the CKD setting. This knowledge coupled with our understanding that PON is a modifiable risk factor, suggest that PON may be an important therapeutic target for combating morbidity and mortality in patients with CKD. 

## 5. Study Limitations

The current study was only sufficiently powered to detect all-cause mortality; thus, these findings will need to be validated in a larger clinical population with sufficient power to detect specific cardiovascular and renal outcomes. Additionally, we only measured the baseline circulating PON-1 protein levels and lactonase activity, therefore we do not know how changes in PON activity over time may influence the outcomes in the setting of CKD. Furthermore, we did not measure HDL, LDL, or other cholesterol values and thus cannot comment on the relationship of these measures of cholesterol status in our study. While the analysis of clinical outcomes was confined to the CKD cohort, it is also important to note that the non-CKD controls were not matched in terms of key demographics such as age or race (albeit that there was 80% similarity in race between CKD and non-CKD patients). Nonetheless, despite these limitations this is the first study that provides information on circulating PON-1 protein levels and a physiologically relevant measure of PON activity (i.e., lactonase activity) in the CKD setting. 

## Figures and Tables

**Table 1:** Clinical characteristics among the participants in the Chronic Renal Insufficiency Standards and Implementation Study (CRISIS).

	*n* (%)	Mean ± SD
Age (year)		69 ± 19
Male	150 (60%)	
White	248 (100%)	
Hispanic/Latino	0 (0%)	
Height (m)		1.7 ± 0.1
Weight (kg)		79 ± 20
BMI (kg/m^2^)		27 ± 8
Systolic Bloood Pressure (mmHg)		135 ± 25
Diastolic Blood Pressure (mmHg)		73 ± 16
Urine protein (mg/dL)		16 ± 46
Creatinine (mg/dL)		2.2 ± 1.7
CKD- Epidemiology Collaboration-eGFR (ml/min per 1.73 m^2^)		30.4 ± 25.9
**Paraoxonase**		
PON Lactonase Activity (pmol/min/mL)		2073.1 ± 850.6
Log PON Lactonase Activity (pmol/min/mL)		7.6 ± 0.4
Median PON Lactonase Activity (High)	123 (50%)	
Median PON Lactonase Activity (Low)	124 (50%)	
PON-1 Protein (ng/mL)		333.4 ± 249.5
Log PON-1 Protein (ng/mL)		5.8 ± 0.7
Median PON-1 Protein (High)	123 (50%)	
Median PON-1 Protein (Low)	124 (50%)	
Adjusted PON Lactonase Activity		6.2 ± 4.6
Log Adjusted PON Lactonase Activity		1.8 ± 0.7
Median Adjusted PON Lactonase Activity (High)	122 (50%)	
Median Adjusted PON Lactonase Activity (Low)	123 (50%)	
**CKD Stage**		
Normal	0 (0%)	
CKD Stage 1	0 (0%)	
CKD Stage 2 (Mild)	9 (4%)	
CKD Stage 3 (Moderate)	103 (42%)	
CKD Stage 4 (Severe)	85 (34%)	
CKD Stage 5 (ESKD)	51 (20%)	
**Type of CKD**		
Diabetic Nephropathy	40 (16%)	
Adult Polycystic Kidney Disease	16 (7%)	
Vascular Hypertension	85 (34%)	
Glomerulonephritis/Vasculitis	33 (13%)	
Pyelonephritis	16 (7%)	
Other	58 (23%)	
**Risk factors/indications**		
Myocardial Infarction	41 (17%)	
Angina	49 (20%)	
Cerebral Vascular Accident	18 (7%)	
Transient Ischemic Accident	21 (8%)	
Diabetes Mellitus	79 (32%)	
Peripheral Vascular Disease	45 (18%)	
Smoking (current)	31 (12%)	
Smoking History	171 (69%)	
**Medication use**		
ACE	96 (39%)	
ARB	61 (25%)	
ACE/ARB	149 (60%)	
β-Blocker	74 (30%)	
Diuretic	113 (46%)	
Statin	140 (56%)	
Aspirin	104 (42%)	
**Endpoints**		
Composite *	167 (67%)	
Mortality	127 (51%)	
Renal Replacement Therapy	66 (27%)	
MACE **	23 (9%)	

Values are expressed as mean ± SD (interquartile range). * Composite endpoint indicates the first occurrence of any of the following events: mortality (cardiovascular or renal death), MACE (myocardial infarction, congestive heart failure, or stroke), and renal replacement therapy. ** MACE indicates major adverse cardiovascular event comprising either myocardial infarction, congestive heart failure, or stroke.

**Table 2:** Unadjusted and adjusted hazard ratio for death at 10 years stratified by median of PON levels.

	PON Activity (pmol/min/mL)		PON Adjusted Activity (pmol/min/ng)		PON-1 Protein (ng/mL)	
Range	≤2073	>2073	≤6.22	>6.22	≤333.4	>333.4
10 years Death, %	72/124	53/123	71/123	53/122	61/124	65/123
Unadjusted Hazard Ratio	1.66 (1.16 to 2.38) **	1	1.51 (1.06 to 2.16) *	1	0.97(0.68 to 1.37)	1
Adjusted HR	1.48 (1.02 to 2.14) *	1	1.55(1.07 to 2.25) *	1	0.99(0.69 to 1.41)	1

Model adjusted for traditional risk factors including age, gender, systolic blood pressure, urine protein (log), myocardial infarction, β-blocker, and angiotensin converting enzyme/angiotensin II receptor blocker. * *p* < 0.05, ** *p* < 0.01.

**Table 3:** Multivariate model for factors that predict the PON lactonase activity in patients with chronic kidney disease.

	Odds Ratio	Std. Error	*p*-Value
Age	0.97	0.011	0.001
BMI	0.94	0.027	0.031

## Supplementary Materials

The following are available online at https://www.mdpi.com/2077-0383/8/7/1034/s1. Table S1: Clinical characteristics among participants in the CRISIS clinical trial stratified by CKD etiology, Table S2: Clinical characteristics among non-CKD healthy controls, Table S3: Unadjusted and adjusted 8-year hazard ratio for death at 8 years stratified by quartile values for circulating PON activity, protein adjusted activity levels, and PON-1 protein. Figure S1A: Comparison of circulating PON-1 protein levels across CKD stages, Figure S1B: Comparison of circulating PON lactonase activity across CKD stages, Figure S1C: Comparison of circulating PON protein adjusted lactonase activity across CKD stages. Figure S2A: Comparison of circulating PON-1 protein levels in CKD etiologies, Figure S2B: Comparison of circulating PON lactonase activity in CKD etiologies, Figure S2C: Comparison of circulating protein adjusted activity in CKD etiologies. Figure S3A: Correlation graph showing relationship between PON lactonase activity and PON-1 protein in non-CKD controls, Figure S3B: Correlation graph showing relationship between PON lactonase activity and PON-1 protein in patients with chronic kidney disease. Figure S4. Western blot analysis of PON1 expression in non-CKD control subjects and patients with CKD.

## Abbreviations

PON	paraoxonase
CKD	chronic kidney disease
CKD-EC-eGFR	CKD-epidemiology collaboration-estimated glomerular filtration rate
ACE	angiotensin converting enzyme
ARB	angiotensin II receptor blocker
MACE	major adverse cardiovascular event
