# Supplementary material for: Circulating Lactonase Activity but Not Protein Level of PON-1 Predicts Adverse Outcomes in Subjects with Chronic Kidney Disease
Source: J Clin Med. 2019 Jul 15;8(7):1034. doi: 10.3390/jcm8071034 (PMC6678354; doi:10.3390/jcm8071034)
Supplement: Supplementary file 1 [file jcm-08-01034-s001.pdf]

Chrysan J. Mohammed
Circulating Lactonase Activity but not Protein Level of PON-1 Predicts Adverse Outcomes in Subjects with Chronic Kidney Disease
Supplementary Data

Table S1. Clinical characteristics among participants in the CRISIS clinical trial stratified by CKD etiology.

	**DM (n = 40)**	**APKD (n = 16)**	**Vascular/HTN (n = 85)**	**GN/Vasculitis (n = 33)**	**Pyelonephritis (n = 16)**	**Other (n = 58)**	***p*-value**
Age (yr)	68 ± 10	57 ± 15	72 ± 10	58 ± 15	49 ± 20	66 ± 14	<0.0001
Male	26 (65%)	3 (19%)	57 (67%)	21 (64%)	4 (25%)	39 (67%)	0.003
White	40 (100%)	16 (100%)	85 (100%)	33 (100%)	16 (100%)	58 (100%)	-
Hispanic/Latino	0 (0%)	0 (0%)	0 (0%)	0 (0%)	0 (0%)	0 (0%)	-
Height (in)	67 ± 3.5	65 ± 3.3	66 ± 3.6	68 ± 4.0	65 ± 4.0	67 ± 3.1	0.11
Weight (lb)	193 ± 34.2	156 ± 24.2	173 ± 36.5	171 ± 40.6	183 ± 45.6	177 ± 33.6	0.02
BMI (kg/m^2)	30.0 ± 5.0	26.6 ± 5.0	27.6 ± 5.6	26.7 ± 5.1	30.1 ± 7.7	28 ± 4.4	0.06
Systolic BP (mmHg)	135 ± 22	130 ± 18	141 ± 20	138 ± 19	127 ± 17	136 ± 20	0.09
Diastolic BP (mmHg)	68 ± 12	77 ± 13	72 ± 12	75 ± 10	77 ± 9	76 ± 12	0.004
Urine Protein (mg/dL)	62 ± 133	21 ± 32	50 ± 98	118 ± 168	35 ± 29	44 ± 66	0.01
Creatinine (mg/dL)	2.9 ± 1.6	2.7 ± 1.3	2.6 ± 1.5	2.9 ± 1.5	2.1 ± 0.7	3.0 ± 2.1	0.35
CKD-EPI-eGFR (ml/min per 1.73 m^2)	27.8 ± 13.1	34 ± 18.5	32 ± 17.0	31 ± 20.0	40 ± 15.0	33 ± 23.2	0.32
**Paraoxonase**							
Activity (pmol/min/mL)	2032.5 ± 563.9	1941.3 ± 516.0	2159.6 ± 738.0	2313.3 ± 533.2	2127.2 ± 393.6	2190.1 ± 746.8	0.41
Log Activity (pmol/min/mL)	7.6 ± 0.29	7.5 ± 0.29	7.6 ± 0.6	7.7 ± 0.24	7.6 ± 0.19	7.6 ± 0.39	0.63
Median Activity (High)	15 (39%)	5 (31%)	44 (52%)	21 (64%)	9 (56%)	29 (50%)	0.23
Median Activity (Low)	23 (61%)	11 (69%)	41 (48%)	12 (36%)	7 (44%)	29 (50%)	-
Protein (ng/mL)	280.4 ± 294.6	427.1 ± 303.8	436.9 ± 307.5	412.8 ± 213.7	430.9 ± 312.5	480 ± 396.3	0.08
Log Protein (ng/mL)	5.3 ± 0.70	5.8 ± 0.71	5.9 ± 0.65	5.9 ± 0.50	5.9 ± 0.58	6.0 ± 0.59	<0.0001
Median Protein (High)	8 (20%)	8 (50%)	46 (55%)	20 (61%)	8 (50%)	32 (56%)	0.002
Median Protein (low)	32 (80%)	8 (50%)	38 (45%)	13 (39%)	8 (50%)	25 (44%)	-
Adjusted† PON Activity	11.4 ± 5.6	5.5 ± 2.8	6.9 ± 5.4	6.6 ± 2.8	6.2 ± 2.7	6.9 ± 4.4	<0.0001
Log Adjusted† PON Activity	2.3 ± 0.64	1.6 ± 0.61	1.7 ± 0.77	1.8 ± 0.45	1.7 ± 0.54	1.7 ± 0.64	0.0004

Median Adjusted Activity (High)	31 (82%)	7 (44%)	36 (43%)	17 (52%)	9 (56%)	22 (39%)	0.001
Median Adjusted Activity (Low)	7 (18%)	9 (56%)	48 (57%)	16 (48%)	7 (44%)	35 (61%)	-
**CKD Stage**							
Mild	0 (0%)	1 (6%)	2 (2%)	2 (6%)	0 (0%)	4 (7%)	
Moderate	15 (38%)	5 (31%)	39 (46%)	12 (36%)	8 (50%)	24 (41%)	
Severe	15 (37%)	6 (38%)	31 (36%)	12 (36%)	7 (44%)	14 (24%)	
ESKD	10 (25%)	4 (25%)	13 (15%)	7 (21%)	1 (6%)	16 (28%)	
**Risk Factors/Indications**							
Myocardial Infarction	6 (15%)	0 (0%)	25 (29%)	1 (3%)	0 (0%)	9 (15%)	<0.001
Angina	9 (22%)	2 (12%)	25 (29%)	0 (0%)	2 (12%)	11 (19%)	0.001
CVA	1 (3%)	1 (6%)	8 (9%)	1 (3%)	2 (12%)	5 (9%)	0.79
TIA	1 (2%)	1 (6%)	11 (13%)	0	2 (12%)	6 (10%)	0.06
Diabetes Mellitus	36 (98%)	1 (6%)	21 (25%)	2 (6%)	3 (19%)	13 (22%)	<0.0001
Peripheral Vascular disease	9 (23%)	1 (6%)	21 (25%)	1 (3%)	2 (12%)	11 (19%)	0.03
Smoking (current)	4 (10%)	2 (12%)	8 (9%)	7 (21%)	0 (0%)	10 (17%)	0.14
Smoking History	29 (73%)	7 (44%)	65 (76%)	23 (70%)	6 (37%)	41 (71%)	0.02
**Medication Use**							
ACE	18 (45%)	8 (50%)	28 (33%)	13 (40%)	9 (56%)	20 (34%)	0.40
ARB	15 (37%)	4 (25%)	18 (21%)	12 (36%)	3 (19%)	9 (15%)	0.10
ACE/ARB	29 (72%)	12 (75%)	45 (53%)	23 (70%)	12 (75%)	28 (48%)	0.04
β-Blocker	9 (22%)	6 (37%)	35 (41%)	8 (24%)	4 (25%)	12 (21%)	0.09
Diuretic	26 (65%)	4 (25%)	47 (55%)	14 (42%)	4 (25%)	18 (31%)	0.001
Statin	27 (67%)	6 (27%)	55 (65%)	22 (67%)	3 (19%)	27 (47%)	0.001
Aspirin	24 (60%)	4 (25%)	45 (53%)	3 (9%)	3 (19%)	25 (43%)	<0.0001
**Endpoints**							
Composite*	28 (70%)	14 (87%)	59 (69%)	24 (73%)	5 (31%)	36 (60%)	0.02
Mortality	23 (57%)	6 (37%)	50 (59%)	14 (42%)	2 (12%)	31 (53%)	0.008
RRT	9 (22%)	10 (62%)	13 (15%)	16 (48%)	3 (19%)	15 (26%)	0.003

MACE**	4 (10%)	2 (12%)	10 (12%)	0 (0%)	2 (12%)	5 (9%)	0.19

Values are expressed as mean ± SD. DM indicates diabetic nephropathy; APKD: adult polycystic kidney disease; vascular/HTN: vascular hypertension; BP: blood pressure; CKD-EC-eGFR: chronic kidney disease-epidemiology collaboration-eGFR; ESKD: end-stage kidney disease; CVA: cerebral vascular accident; TIA: transient ischemic accident; ACE: angiotensin converting enzyme; ARB: angiotensin II receptor blocker. * Composite endpoint indicates the first occurrence of any of the following events: mortality (cardiovascular or renal death), MACE (myocardial infarction, congestive heart failure, or stroke), and renal replacement therapy. **MACE indicates major adverse cardiovascular event comprising either myocardial infarction, congestive heart failure, or stroke.

Table S2. Clinical characteristics among non-CKD healthy controls

	**N (%)**	**Mean ± SD**
Age (yr)		30 ± 10
Male	15 (45%)	
White	26 (81%)	
Black	1 (3%)	
Asian	3 (9%)	
Other	2 (6%)	
Height (m)		1.7 ± 1.0
Weight (kg)		80 ± 19
BMI (kg/m^2)		28.5 ± 7.0
Systolic Bloood Pressure (mmHg)		117 ± 10
Diastolic Blood Pressure (mmHg)		74 ± 7
Creatinine (mg/dL)		0.89 ± 0.21
CKD- Epidemiology Collaboration-eGFR (ml/min per 1.73 m^2)		102.7 ± 16.9
**Paraoxonase**		
PON Lactonase Activity (pmol/min/mL)		3514.9 ± 745.1
PON-1 Protein (ng/mL)		682.9 ± 214.0

Values are expressed as mean ± SD.

Table S3. Unadjusted and adjusted 8-year hazard ratio for death at 8 years stratified by quartile values for circulating PON activity, protein adjusted activity levels, and PON-1 protein.

		**PON Activity (pmol/min/mL)**		
	**Q 4**	**Q3**	**Q2**	**Q1**
Range	≥2576	2073–2576	1732–2073	<1732
Unadjusted HR	1	1.12 (0.64–1.93) p = 0.70	1.66 (0.99–2.78) p = 0.05	1.76 (1.04–2.97) *p = 0.03
Adjusted HR	1	1.01 (0.57–1.78) p = 0.97	1.24 (0.74–2.09) p = 0.41	1.92 (1.12–3.29) *p = 0.02
**8-year Death (%)**	25/62 = 40.3	26/62 = 41.9	35/61 = 57.4	34/62 = 54.8
		**Protein Adjusted PON Activity (pmol/min/ng)**		
	**Q 4**	**Q3**	**Q2**	**Q1**
Range	≥8.99	6.22–8.99	4.47–6.22	<4.47
Unadjusted HR	1	1.01 (0.59–1.75) p = 0.96	1.24 (0.72–2.12) p = 0.44	1.71 (1.03–2.85) *p=0.04
Adjusted HR	1	0.83 (0.48-1.45) p = 0.51	1.16 (0.67–2.00) p = 0.60	1.51 (0.89–2.55) p = 0.13
**8-year Death (%)**	25/62 = 40.3	27/61 = 44.3	29/61 = 47.5	38/61 = 62.3
		**PON-1 Protein (ng/mL)**		
	**Q 4**	**Q3**	**Q2**	**Q1**
Range	≥472.35	333.4–472.35	226.85–333.4	<226.85
Unadjusted HR	1	0.69 (0.42–1.14) p = 0.14	0.80 (0.49–1.30) p = 0.36	0.81 (0.49–1.33) p = 0.40
Adjusted HR	1	0.63 (0.38–1.05) p = 0.08	0.72 (0.43–1.18) p = 0.19	0.89 (0.54–1.46) p = 0.64
**8-year Death (%)**	35/62 = 56.5	28/62 = 45.2	29/61 = 47.5	29/62 = 46.8

Model adjusted for traditional risk factors including age, gender, systolic blood pressure, urine protein (log), myocardial infarction, β-blocker, and ACE/ARB. HR indicates hazard ratio; ACE, angiotensin converting enzyme; ARB, angiotensin II receptor blocker. *$p < 0.05$, **$p < 0.01$.

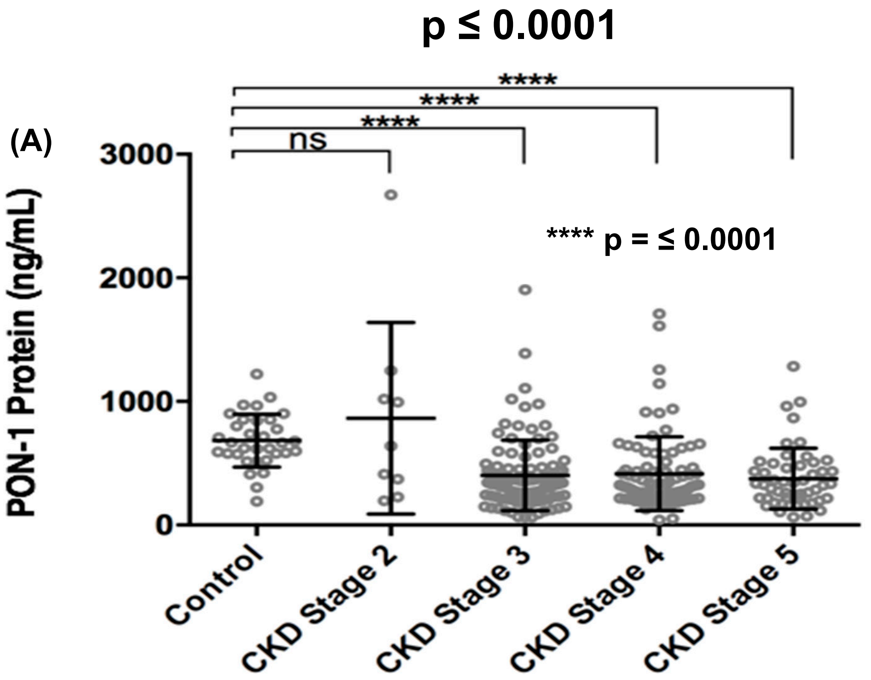
p ≤ 0.0001
(A)
ns
****
****
****
**** p = ≤ 0.0001
PON-1 Protein (ng/mL)
3000
2000
1000
0
Control
CKD Stage 2
CKD Stage 3
CKD Stage 4
CKD Stage 5

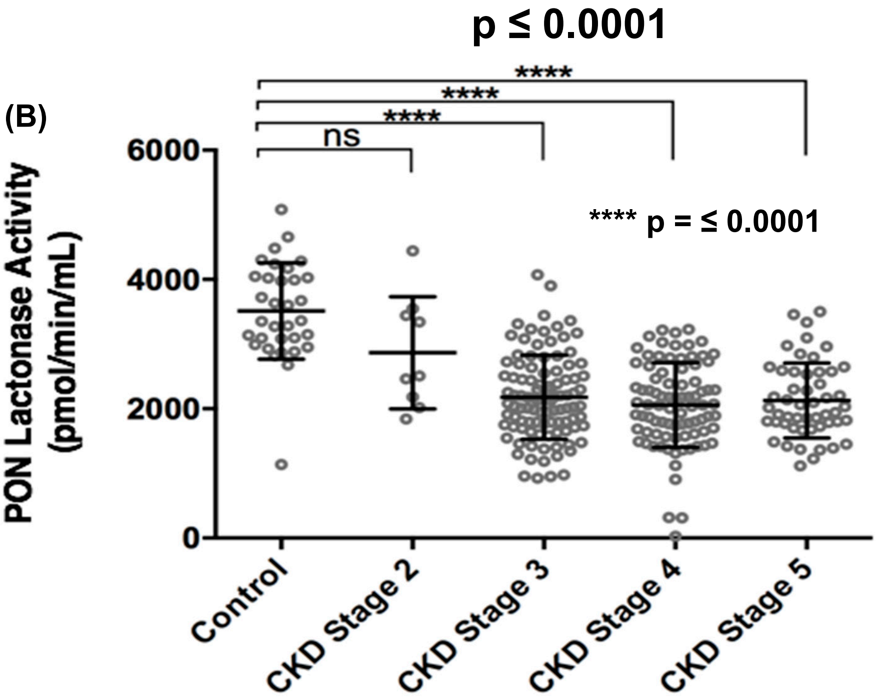
p ≤ 0.0001
(B)
ns
****
****
****
**** p = ≤ 0.0001
PON Lactonase Activity (pmol/min/mL)
6000
4000
2000
0
Control
CKD Stage 2
CKD Stage 3
CKD Stage 4
CKD Stage 5

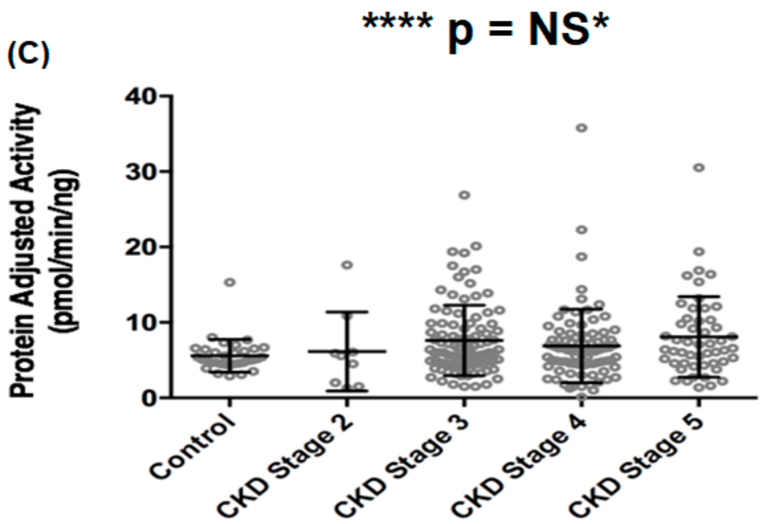

Figure 1. (A) Comparison of circulating PON-1 protein levels across CKD stages. NS indicates not significant. **(B)** Comparison of circulating PON lactonase activity across CKD stages. NS indicates not significant. **(C)** Comparison of circulating PON protein adjusted lactonase activity across CKD stages. Protein adjusted lactonase activity is the ratio of PON activity and PON-1 protein. NS indicates not significant.

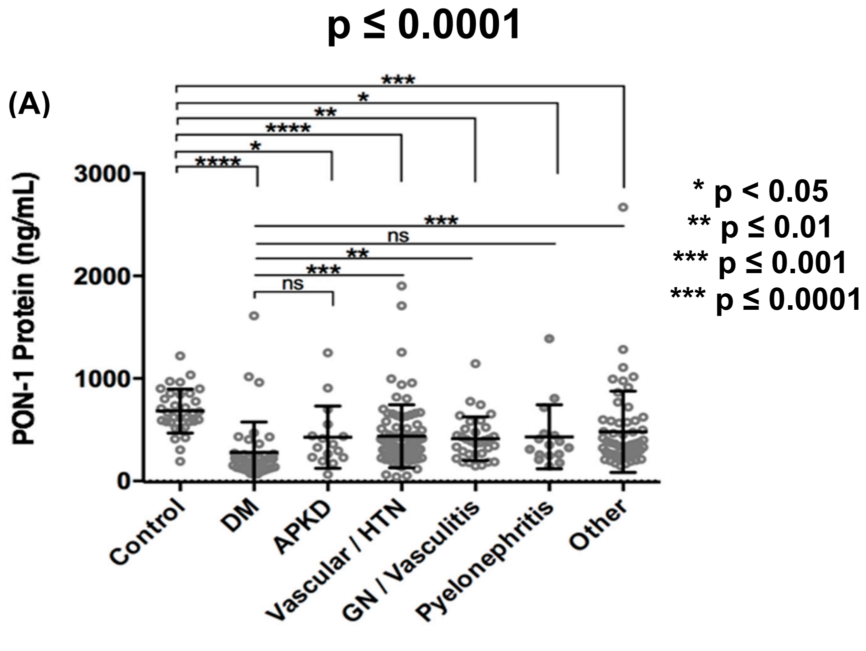

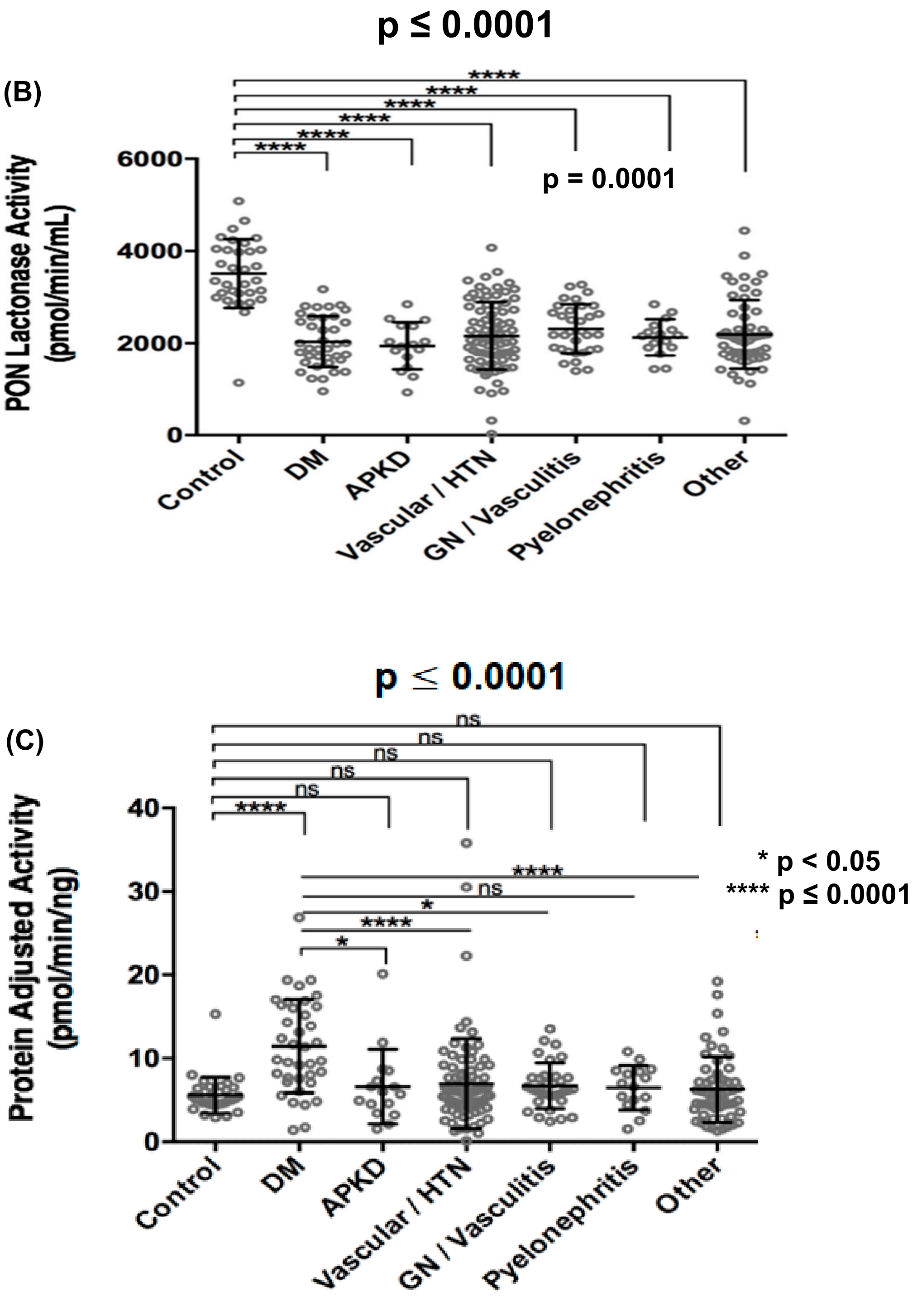

Figure 2. (A) Comparison of circulating PON-1 protein levels in CKD etiologies. DM indicates diabetes nephropathy; APKD: adult polycystic kidney disease; vascular/HTN: vascular hypertension; GN/VAS: glomerulonephritis vasculitis; NS: not significant. **(B)** Comparison of circulating PON lactonase activity in CKD etiologies. DM indicates diabetes nephropathy; APKD: adult polycystic kidney disease; vascular/HTN: vascular hypertension; GN/VAS: glomerulonephritis vasculitis. **(C)** Comparison of circulating protein adjusted activity in CKD etiologies. DM indicates diabetes nephropathy; APKD: adult polycystic kidney disease; vascular/HTN: vascular hypertension; GN/VAS: glomerulonephritis vasculitis; NS: not significant.

(A)

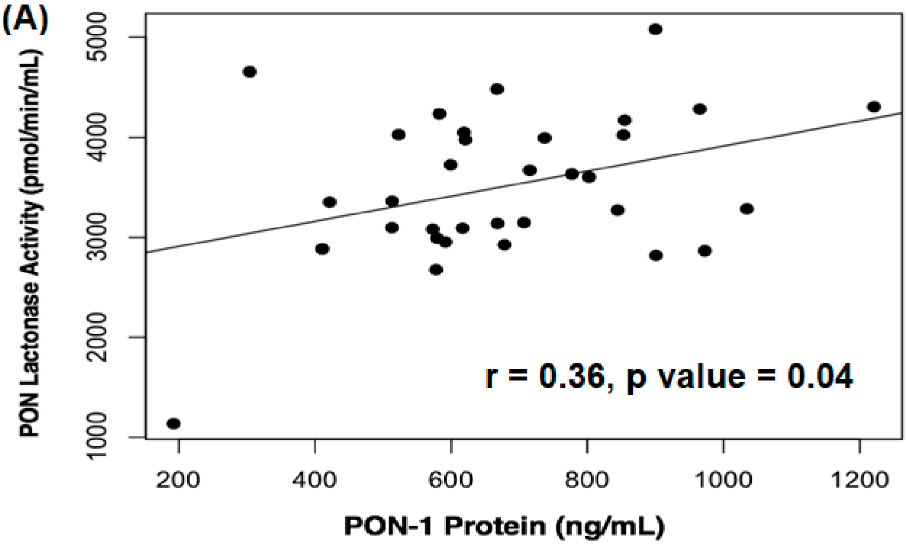

(B)

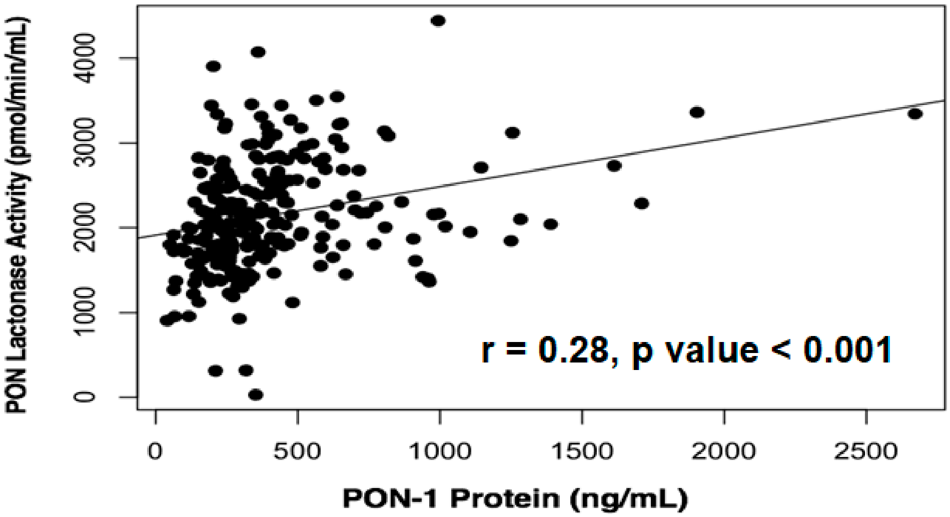

Figure S3. (A). Correlation graph showing relationship between PON lactonase activity and PON-1 protein in non-CKD controls. **(B)** Correlation graph showing relationship between PON lactonase activity and PON-1 protein in patients with chronic kidney disease.

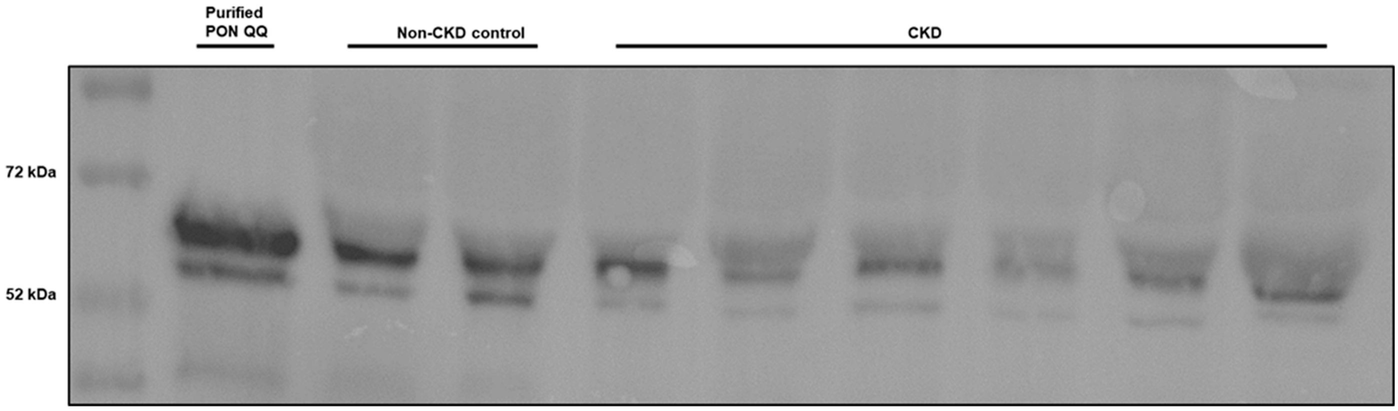

Figure S4. Western blot analysis of PON1 expression in non-CKD control subjects and patients with CKD
